# Retrospective analysis of risk factors for deep infection in lower limb Gustilo–Anderson type III fractures

**DOI:** 10.1186/s10195-020-00549-5

**Published:** 2020-07-18

**Authors:** Taku Ukai, Kosuke Hamahashi, Yoshiyasu Uchiyama, Yuka Kobayashi, Masahiko Watanabe

**Affiliations:** Department of Orthopaedic Surgery, Surgical Science, Tokai University School of Medicine, 143 Shimokasuya, Isehara, Kanagawa 259-1193 Japan

**Keywords:** Open fracture, Infection, Soft-tissue reconstruction, Drug-resistant bacteria

## Abstract

**Background:**

Open fractures are among the most severe injuries observed in orthopedic patients. Treating open fractures is difficult because such patients with infections may require multiple operations and amputations. Furthermore, only a few studies have focused on antibiotic prophylaxis in open fractures and evaluated how to cover lost soft tissue to increase the success rate of reconstruction. We evaluated the risk factors for deep infection in lower limb Gustilo–Anderson (G–A) type III fractures.

**Materials and methods:**

This retrospective study investigated patients who underwent surgical procedures for lower limb G–A type III fractures between January 2007 and January 2017 at our institution. We enrolled 110 patients with 114 lower limb G–A type III fractures (77 G–A type IIIA fractures and 37 G–A type IIIB fractures) who were followed up for at least 2 years. We compared patients presenting infections with those without infections by assessing the following factors: severe contamination, diabetes, smoking, Injury Severity Scale, segmental fracture, location of fracture, G–A classification, damage control surgery, methods of surgery, timing of fixation, combination of antibiotics used, duration of antibiotic prophylaxis, timing of wound closure, and soft-tissue reconstruction failure.

**Results:**

Eighteen fractures presented deep infections. Compared with patients without infections, patients developing infections differed significantly in terms of severe contamination (*P* < 0.01), G–A classification (*P* < 0.01), duration of antibiotic prophylaxis (*P* < 0.01), timing of wound closure (*P* < 0.01), and incidence of soft-tissue reconstruction failure (*P* < 0.01). Skin grafting was associated with significantly higher failure rates than muscle and free flap reconstructions (*P* = 0.04). Treatment with antibiotics was significantly longer in patients with drug-resistant bacterial infections than in those without infections (*P* < 0.01).

**Conclusion:**

Early flaps rather than skin grafting should be used to cover G–A type IIIB fractures, because skin grafting resulted in the highest failure rate among soft-tissue reconstructions in open fractures. Longer duration of antibiotic use had a significant impact not only on deep infection rates but also on the presence of drug-resistant bacteria. These findings suggest that prolonged use of antibiotics should be avoided in cases of open fractures.

**Level of evidence:**

Level IV retrospective observational study.

## Introduction

G–A type III open fractures have a high infection rate [1–3]. Once deep infection or osteomyelitis has occurred, patients may require multiple operations and may develop significant dysfunction at the site of injury. Risk factors for deep infection include diabetes [4–8] and smoking [9, 10]. Specifically, G–A type III open fractures are associated with severe soft-tissue injuries [1]. In cases where open fractures are complicated by massive loss of soft tissue, patients often require soft-tissue reconstruction. Although it is desirable to manage an open fracture by restoration with a soft-tissue cover as quickly as possible, this is often complicated by the requirement for cooperation with plastic surgeons. Therefore, it is essential to determine when to cover injuries that have lost soft tissue and to choose the optimal methodology. Lack et al. reported that the infection rate was significantly lower in patients in whom the wound was closed within 5 days of the injury [7]. Nevertheless, only a limited number of reports have evaluated how to cover lost soft tissue to increase the success rate of reconstruction [11, 12]. While treatment with antibiotics is recommended 24–72 h after wound closure [13], only a few reports have focused on antibiotic prophylaxis in open fractures [8, 13–15]. Open fractures may prolong the need for antibiotic therapy because they are frequently complicated by pneumonia, urinary tract infections, use of an artificial respirator, as well as several other conditions [16]. The purpose of this study is to identify factors that increase the risk of deep infection in lower limb G–A type III fractures.

## Materials and methods

### Study design and population

Patients with open fractures who underwent surgery from 2007 to 2017 were retrospectively investigated. The patient’s mean age was 44.5 years (range 18–84 years). Overall, 81 patients (84 total fractures) were men, and 29 patients (30 total fractures) were women. Eighteen fractures (15.8%) were diagnosed with a concomitant deep infection, and 3 resulted in amputation as a consequence of the infection. The mechanisms of injury were as follows: traffic accidents, 88 fractures; falls, 16 fractures; and other trauma, 10 fractures. The mean follow-up period was 37 months (range 24–119 months), and the mean Injury Severity Score (ISS) was 13.7 (range 9–34).

**Fig. 1:**
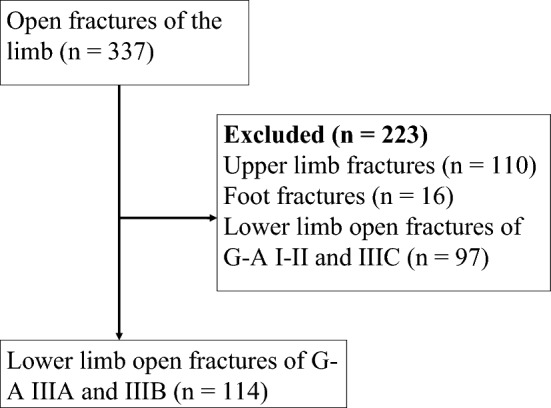
Flow diagram showing patient identification and exclusion

The inclusion criteria were patients with lower limb open fractures of G–A type III who were followed up for at least 2 years after the surgical procedure. Exclusion criteria included open fractures of the upper limb and foot, closed fractures, and open fractures of G–A types I, II, and IIIC (Fig. 1).

### Fractures

Fractures were classified as follows: 77 G–A type IIIA fractures and 37 G–A type IIIB fractures. The location of the fractures was as follows: femur in 21 fractures, tibia in 54 fractures, and ankle in 39 fractures. We evaluated open fractures using the modified G–A classification. We evaluated the soft-tissue condition rather than the length of the wound. We defined fractures without severe contamination and extensive soft-tissue damage and those for which we could close the wounds as G–A type IIIA. Fractures with extensive soft-tissue damage and those for which we could not close the wounds were defined as G–A type IIIB (Table 1). We defined severe contamination as a wound that was contaminated with mud or sand.

**Table 1:** Modified Gustilo–Anderson classification

Type	I	II	IIIA	IIIB	IIIC
Wound size	≤ 1 cm	1–10 cm	About 5–10 cm	About 10 cm or longer	About 10 cm or longer
Soft-tissue damage	Minimal	Minimal	Minimal or moderate	Severe	Severe
Contamination	Clean	Clean or moderate	Clean or moderate	Moderate or severe	Moderate or severe
Wound closure	Primary suture	Primary suture	Primary suture	Soft-tissue coverage	Primary suture or soft-tissue coverage
Revascularization	Not needed	Not needed	Not needed	Not needed	Required

### Criteria for deep infection

Deep infection was diagnosed using recommendations from the Centers for Disease Control and Prevention [17]. In patients who were diagnosed with a deep infection, bacterial cultures were performed before debridement, during debridement, or both. Deep infection included a documented surgical site infection with bone involvement. The present study did not include patients with superficial infections. Pin tract infection not requiring surgery was also excluded.

### Treatment strategy

The treatment strategy for open fractures adopted at the institution was as follows: open reduction and internal fixation (ORIF) was performed on the day of admission in patients with G–A type IIIA femur, tibia, and ankle fractures. Patients who had comminuted fractures, such as pilon and bicondylar tibial plateau fractures of G–A type IIIA, underwent external fixation as damage control surgery on the day of admission, and ORIF was performed after the soft-tissue swelling improved, as assessed by presence of skin wrinkling. Patients with open fractures of G–A type IIIB underwent external fixation as damage control surgery on the day of admission, and ORIF and soft-tissue reconstruction were performed after the soft-tissue swelling improved. Patients who presented with severe head, chest, or abdominal injury or had contraindications for surgery had their wounds washed and their fractures fixed with splints in treatment rooms; they underwent ORIF or external fixation once their general condition improved.

### Soft-tissue reconstruction

The mean time required for soft-tissue reconstruction was 13.7 days (range 0–31 days) for G–A type IIIB fractures. Prior to performing soft-tissue reconstruction, 20 fractures were treated with wet dressing and 17 patients were treated with negative-pressure wound therapy (NPWT). Twenty-six patients were treated with skin grafting only (femur: 2, tibia: 17, ankle: 7), 5 patients were treated with a muscle flap (tibia: 3, ankle: 2), and 6 patients with a free flap (femur: 1, tibia: 4, ankle: 1). Skin grafting was performed as the only soft-tissue reconstruction method, and we attempted to completely cover the bones with soft tissue when performing skin grafting. During the skin grafting procedure, we meticulously checked whether the wounds were sufficiently covered with soft tissue and attempted to preserve the periosteum of the bone. Overall, 8 of 26 patients who received skin grafting underwent repeat operation. Of these eight patients, six were diagnosed with deep infections. Failure of soft-tissue reconstruction was considered in patients who underwent additional soft-tissue reconstruction after the first intervention.

### Antibiotics and culture

A first-generation cephalosporin was administered for 96 fractures, while a combination of cephalosporin and aminoglycoside was administered for 18 fractures. For patients under 80 kg, we used 1 g of a first-generation cephalosporin twice a day; for patients weighing over 80 kg, we used 2 g of a first-generation cephalosporin twice a day. Aminoglycoside dosage was accompanied by therapeutic drug monitoring. In our institution, antibiotic withdrawal is decided by evaluating patients’ fever, laboratory data (white blood cells and C-reactive protein), and wound discharge. The mean duration of antibiotic treatment was 11.9 days (range 2–34 days), and this period included only antibiotic prophylaxis. The antibiotic treatment duration was defined as the period of continuous use of antibiotics from the day of admission. This period did not include therapeutic antibiotic administration. Bacteria were identified in all patients diagnosed with a deep infection. The specific bacteria detected were as follows: methicillin-resistant *Staphylococcus aureus* (MRSA) in nine fractures, methicillin-resistant staphylococci (MRS) in one fracture, and other bacteria in eight fractures (Table 2).

**Table 2:** Details of culture tests

Isolated organism	Number
Methicillin-resistant *Staphylococcus aureus*	9
Methicillin-resistant staphylococci	1
Other	8

### Statistical analyses

We evaluated age, body mass index, ISS, timing of fixation, duration of antibiotic prophylaxis, and timing of wound closure using the Mann–Whitney *U* test. We used Fisher’s exact test to evaluate severe contamination, diabetes, smoking status, segmental fracture, location of fracture, G–A classification, damage control surgery, methods of surgery, combinations of antibiotics, and soft-tissue reconstruction failure. We divided patients further into two groups: patients with resistant bacterial infections (10 fractures), and patients without resistant bacterial infections (8 fractures). The Mann–Whitney *U* test was used to compare patients with and without antibiotic-resistant bacteria by evaluating the duration of antibiotic use and hospital stay. All tests were performed with a significance level of *P* < 0.05.

## Results

The comparison of fractures with and without deep infection revealed significant differences in terms of the presence of severe contamination (with infection 11.1% [2/18] versus without infection 4.2% [4/96], *P* < 0.01), G–A fracture classification (infection rates: IIIA 7.8% [6/77] versus IIIB 32.4% [12/37], *P* < 0.01), duration of antibiotic prophylaxis (with infection: 14.1 days versus without infection: 11.2 days, *P* < 0.01), timing of wound closure (with infection: 8.8 days versus without infection: 4 days, *P* < 0.01), and soft-tissue reconstruction failure (failure rate: 21.6%, *P* < 0.01) (Table 3). Fisher’s exact test also revealed that the occurrence of skin graft failure was significantly higher than that of muscle and free flap failure (failure rates: skin graft, 30.8% [8/26] versus muscle flap 0% [0/5], free flap 0% [0/6], *P* = 0.04).

**Table 3:** Comparison of fractures with and without infection

Valuable	With infection	Without infection	*P* value
Age (years)	47.8 (± 19.4)	43.1 (± 19.7)	0.41
Body mass index (kg/m^2^)	24.4 (± 4.8)	23 (± 4.9)	0.18
Severe contamination	5	5	< 0.01
Diabetes	2	3	0.13
Smoking	10	46	0.61
Injury Severity Scale	16.8 (± 7.0)	13.2 (± 7.2)	0.06
Segmental fracture	2	4	0.2
Location of fracture			0.5
Femur	4	17	
Tibia	10	44	
Ankle	4	35	
G–A classification			< 0.01
IIIA	6	71	
IIIB	12	25	
Damage control surgery	13/18	58/96	0.44
Methods of surgery			0.11
Nail	6	35	
Plate	7	42	
External fixation	3	4	
Pinning	2	15	
Timing of fixation (days)	9.1 (± 6.6)	8.5 (± 7.3)	0.3
Combination of antibiotics			0.3
First-generation cephalosporin	16	80	
First-generation cephalosporin + aminoglycoside	3	15	
Antibiotic duration (days)	14.1 (± 7.7)	11.2 (± 5.9)	< 0.01
Timing of wound closure (days)	8.8 (± 9.4)	4 (± 8.0)	< 0.01
Soft-tissue reconstruction failure	6	2	< 0.01

The Mann–Whitney *U* test revealed that the duration of antibiotic prophylaxis was significantly longer in patients who had drug-resistant bacterial infections than in those who did not (19.8 versus 11.2 days, *P* < 0.01). Hospital stay duration was not significantly different (*P* = 0.73) between patients who had resistant bacteria (45.2 days) and those who did not (35.4 days).

## Discussion

The findings of the comparison between the infection and noninfection groups revealed that severe contamination, G–A classification, duration of antibiotic prophylaxis, the timing of wound closure, and soft-tissue reconstruction failure had a substantial impact on deep infections. Skin grafts alone had significantly higher failure rates than muscle and free flap reconstructions. The antibiotic prophylaxis duration was significantly longer in patients who had drug-resistant bacterial infections than in those who did not.

Various factors related to the rates of deep infection in open fractures have been reported previously [4–10], including associated soft-tissue injury, fracture type, and treatment strategy. In particular, some types of open fracture such as gunshot and farmyard injuries and segmental fractures may influence deep infections. In this study, although there were no cases of gunshot or farmyard injuries, six patients had segmental fractures. Unlike other fractures, segmental fractures frequently entail severe soft-tissue injuries that induce deep infection. However, there were no significant differences between patients with and without infection (Table 3). Regarding G–A classification, G–A type III fractures are reported to have a higher infection rate than G–A types I and II [18], with G–A type IIIB specifically having the highest rate of infection [17–19]. This suggests that G–A type III fractures frequently complicate severe soft-tissue injury, and as such, it is important to cover the skin injury as soon as possible. We found significant differences in the timing of primary wound closure and soft-tissue reconstruction failure, defined as multiple soft-tissue reconstruction surgeries and the resultant deep infection rate after open fracture. The timing of wound closure was longer for G–A type IIIB fractures than for G–A type IIIA fractures because G–A type IIIB fractures require meticulous soft-tissue reconstruction. Open fractures should be closed as soon as possible. Gopal et al. [12] stated that G–A type IIIB fractures should be covered in less than 72 h. Most G–A type IIIB fractures require flaps; however, such surgery is long and entails the risk of flap necrosis. Thus, we performed skin grafting when bones were covered with muscle and soft tissue. The G–A classification is used for evaluating open fractures worldwide, and we classified open fractures according to this classification in this study. However, the G–A classification cannot evaluate muscle and soft-tissue damage. These factors may have affected infection, and our grouping of open fractures may have been inappropriate. Thus, we are evaluating open fractures prospectively by evaluating these factors.

According to British Association of Plastic, Reconstructive and Aesthetic Surgeons guidelines [20], open fractures should be covered within 5–7 days after injury. In this study, the timing of wound closure of the infection group was longer than that recommended, as some patients required NPWT to improve their wound conditions. Previous metaanalyses have concluded that NPWT not only reduces the infection rate but also reduces flap necrosis and flap revision rates [21, 22]. By contrast, the WOLLF trial demonstrated that there was no improvement in wounds with NPWT and that delays in covering the fracture should be avoided [23]. In this study, we valued the soft-tissue condition more than the interval between soft-tissue reconstruction and injury so as to improve the soft-tissue condition. However, delayed skin closure may affect infection rates. The results suggest that it is more important to cover a wound within 7 days than to use NPWT for over 7 days, so as to reduce infection.

Mathews et al. [24] reported that patients who underwent multiple reconstructive surgeries had higher infectious complication rates than those who underwent single-stage orthoplastic fixation and coverage (34.6% versus 4.2%). Soft-tissue reconstruction failure is almost synonymous with the development of deep infection. We believe that soft-tissue reconstruction failure leads to the development of infection. As the skin prevents bacteria on our bodies from infiltrating the wound and soft-tissue reconstruction failure prolongs the period during which the skin barrier is missing, bacteria may easily infiltrate wounds. In this study, we meticulously checked wounds and attempted to preserve the bone periosteum. We covered bone with soft tissues sufficiently before performing skin grafting. However, skin grafting had the highest failure rate (30.8%) among the methods of soft-tissue reconstruction. This suggests that skin grafting is insufficient for treating G–A type IIIB fractures. Although skin grafting is a less complex procedure to perform than a muscle flap or free flap intervention, it frequently fails at locations with thin soft tissue; For example, it has been reported that a fracture of the tibia has a 244% increased risk of a deep infection compared with a nontibial fracture [25]. In our study, 8 of 26 patients who underwent skin grafting required reoperation, and 6 of 8 patients were diagnosed with a deep infection. Typically, at our hospital, surgeons choose a skin graft when bones and tendons are sufficiently covered by muscle but choose muscle and free flap procedures when bones and tendons are not sufficiently covered by muscle tissue, in accordance with established institutional criteria. However, we may unintentionally choose soft-tissue reconstruction inappropriately instead of a muscle flap or free flap, because these procedures require longer operation times and are more difficult procedures than skin grafting.

There are a limited number of reports that provide sufficient evidence regarding the combination of antibiotics to be used or their route and duration of administration in open fractures. Redfern et al. compared a combination of cefazolin and gentamicin with piperacillin/tazobactam in G–A type III fractures [26]. They reported that there was no significant difference between treatments and the rate of infection. Lloyd et al. reported that the infection rate was slightly decreased when antibiotics specifically targeting Gram-negative bacteria were selected; however, there were no significant differences in the rate of osteomyelitis [27]. Based on these results, the authors recommended cefazolin or clindamycin in open fractures. However, other authors have recommended cefazolin plus aminoglycoside or ampicillin plus sulbactam [28–30]. In the present study, we used cefazolin plus aminoglycoside to cover Gram-negative bacteria when the wound was particularly contaminated. Interestingly, there was no significant difference between the cephalosporin group and the cephalosporin plus aminoglycoside group. This may be due to the retrospective nature of our study and that the combination of antibiotics used was based on the individual clinician’s discretion. Therefore, an extensive investigation to determine the most efficacious combination of antibiotics is needed in future studies.

Regarding the duration of antibiotic use, Hoff et al. recommended 72-h administration of antibiotics within 24 h of wound closure in G–A type III [13]. Long-term antibiotic use can lead to drug-resistant bacteria [14, 31, 32]; in our case, drug-resistant bacteria were detected in over half of the patients with infections (Table 2). The occurrence of drug-resistant bacteria was also significantly higher with prolonged antibiotic administration (19.8 versus 11.6 days). This finding suggests that the duration of antibiotic therapy had a significant impact on deep infection. Our findings also suggest that long-term antibiotic administration should be avoided to decrease the rate of drug-resistant bacterial infections.

This study has several limitations. First, multiple factors affect the occurrence of deep infection after open fractures, such as degree of soft-tissue injury and timing of antibiotic therapy. However, given the retrospective nature of this study, we could not evaluate these factors. Second, we could not evaluate potential risk factors associated with open fractures using logistic regression analysis due to the lack of an adequate sample size. Thus, we are currently gathering data related to open fractures to increase the sample size for a future analysis. Third, we could not control the combination of antibiotics used or their duration of administration. Fourth, we could not evaluate contamination of the wound quantitatively. Previous studies have reported that a contaminated wound increases the deep infection rate [25, 26, 33]. Nevertheless, there are no standard criteria for determining whether a wound is severely contaminated with mud, sand, or seawater; therefore, additional experimentation is needed in the future to evaluate these criteria. Fifth, we used the G–A classification to evaluate open fractures, which is widely accepted by many researchers. Nevertheless, it has been pointed out that, despite the fact that G–A type IIIB includes a wide range of open injuries, there are only two categories of severe fractures (G–A types IIIB and IIIC), and the reliability and reproducibility of this classification have been demonstrated to be suboptimal. The Classification and Outcomes Committee of the Orthopaedic Trauma Association (OTA) was created to overcome the problems with the G–A classification. Agel et al. [34] reported that the OTA classification demonstrated moderate to excellent interobserver reliability. This classification consists of five factors: skin injury, muscle injury, arterial injury, contamination, and bone loss. Nevertheless, we could not evaluate muscle and contamination efficiently because of the retrospective nature of this study. Sixth, antibiotic withdrawal was at the discretion of each orthopedic surgeon, and this may have affected the results.

In conclusion, we believe that skin grafting should not be applied to G–A type IIIB fractures while early flap coverage should be the preferred option. Antibiotic treatment duration had a significant impact not only on deep infection rates but also on the presence of drug-resistant bacteria. These data suggest that prolonged antibiotic use should be avoided in cases of open fractures.

## Data Availability

Not applicable.

## Abbreviations

G–A: Gustilo–Anderson
ORIF: Open reduction and internal fixation
MRSA: Methicillin-resistant *Staphylococcus aureus*
MRS: Methicillin-resistant staphylococci
